# 3-(4-Methoxy­benzo­yl)propionic acid

**DOI:** 10.1107/S1600536808034508

**Published:** 2008-10-25

**Authors:** Sajid Ali, Nasim Hassan Rama, Ghulam Qadeer, Ales Ruzicka

**Affiliations:** a Department of Chemistry, Quaid-i-Azam University, Islamabad 45320, Pakistan; b Department of General and Inorganic Chemistry, Faculty of Chemical Technology, University of Pardubice, Nam. Cs. Legii’ 565, 53210 Pardubice, Czech Republic

## Abstract

In the crystal of the title compound, C_11_H_12_O_4_, inversion dimers arise from pairs of intermolecular O—H⋯O hydrogen bonds and C—H⋯O bonds further consolidate the packing. There is also a C—H⋯π contact between the benzene ring and the methyl­ene group.

## Related literature

For general background, see: Hashem *et al.* (2007 ▶); Husain *et al.* (2005 ▶). For bond-length data, see: Allen *et al.* (1987 ▶).
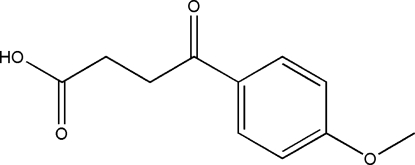


## Experimental

### 

#### Crystal data

C_11_H_12_O_4_
*M* *_r_* = 208.21
Monoclinic, 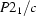

*a* = 5.0511 (3) Å
*b* = 10.0219 (7) Å
*c* = 20.0840 (12) Å
β = 90.107 (6)°
*V* = 1016.67 (11) Å^3^
*Z* = 4
Mo *K*α radiation
μ = 0.10 mm^−1^
*T* = 150 (1) K
0.20 × 0.18 × 0.13 mm

#### Data collection

Bruker–Nonius KappaCCD area-detector diffractometer
Absorption correction: integration (Coppens, 1970 ▶) *T*_min_ = 0.979, *T*_max_ = 0.987
8320 measured reflections
2236 independent reflections
1662 reflections with *I* > 2σ(*I*)
*R*_int_ = 0.048

#### Refinement

*R*[*F* ^2^ > 2σ(*F* ^2^)] = 0.048
*wR*(*F* ^2^) = 0.112
*S* = 1.13
2236 reflections
136 parameters
H-atom parameters constrained
Δρ_max_ = 0.18 e Å^−3^
Δρ_min_ = −0.20 e Å^−3^

### 

Data collection: *COLLECT* (Hooft, 1998 ▶); cell refinement: *COLLECT* and *DENZO* (Otwinowski & Minor, 1997 ▶); data reduction: *COLLECT* and *DENZO*; program(s) used to solve structure: *SIR92* (Altomare *et al.*, 1994 ▶); program(s) used to refine structure: *SHELXL97* (Sheldrick, 2008 ▶); molecular graphics: *PLATON* (Spek, 2003 ▶); software used to prepare material for publication: *SHELXL97*.

## Supplementary Material

Crystal structure: contains datablocks I, global. DOI: 10.1107/S1600536808034508/hk2556sup1.cif

Structure factors: contains datablocks I. DOI: 10.1107/S1600536808034508/hk2556Isup2.hkl

Additional supplementary materials:  crystallographic information; 3D view; checkCIF report

## Figures and Tables

**Table 1:** Hydrogen-bond geometry (Å, °)

*D*—H⋯*A*	*D*—H	H⋯*A*	*D*⋯*A*	*D*—H⋯*A*
O2—H2⋯O1^i^	0.82	1.81	2.628 (3)	173
C6—H6⋯O3^ii^	0.93	2.34	3.247 (3)	164
C11—H11*B*⋯O4^iii^	0.96	2.60	3.328 (3)	133
C3—H3*B*⋯*Cg*1^iv^	0.97	2.74	3.591 (3)	146

Symmetry codes: (i) 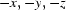; (ii) 
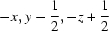
; (iii) 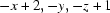; (iv) 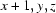. *Cg*1 is the centroid of the phenyl ring.

**Fig. 1.**
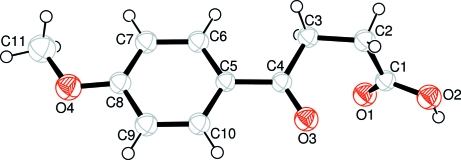
The molecular structure of the title molecule, with the atom-numbering scheme.

**Fig. 2.**
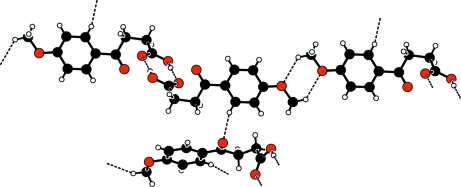
A partial packing diagram. Hydrogen bonds are shown as dashed lines.

**Fig. 3.**
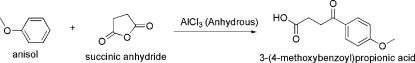
The formation of the title compound.

## supplementary crystallographic information

### Comment

Benzoyl propionic acids are important intermediates in heterocyclic chemistry and have been used for the synthesis of various biologically active five -membered heterocyles such as butenolides, pyrrolones (Husain *et al.*, 2005), oxadiazoles and triazoles (Hashem *et al.*,  2007). In view of the versatility of these compounds, we synthesized the title compound and reported herein its crystal structure.

In the title compound (Fig. 1), the bond lengths (Allen *et al.*, 1987) and angles are within normal ranges. O3, O4, C2, C3 and C4 atoms are 0.067 (3), -0.003 (3), -0.163 (4), -0.013 (3) and 0.016 (3) Å away from the phenyl plane, respectively.

In the crystal structure, intermolecular O-H···O and C-H···O hydrogen bonds (Table 1) link the molecules (Fig. 2), in which they may be effective in the stabilization of the structure. There also exist a C—H···π contact (Table 1) between the phenyl ring and the methylene group.

### Experimental

The title compound was synthesized by the condensation of succinic anhydride (2 g, 20 mmol) with anisol (10 ml) in the presence of alumium chloride (6 g, 42 mmol). The reaction mixture was refluxed for 4 h. After completion of the reaction, excess solvent (anisol) was removed by steam distillation. The resultant solid product was purified by dissolving it in sodium hydroxide solution (5%, *w*/*v*), filtering followed by addition of hydrochloric acid. The obtained solid mass was filtered, washed with cold water, dried and crystallized from methanol (yield; 55%, m.p. 419-420 K)

### Refinement

H atoms were positioned geometrically, with O-H = 0.82 Å (for OH) and C-H = 0.93, 0.97 and 0.96 Å for aromatic, methylene and methyl H, respectively, and constrained to ride on their parent atoms with U_iso_(H) = 1.2U_eq_(C,O).

### Crystal data

**Table:**

C_11_H_12_O_4_	*F*(000) = 440
*M**_r_* = 208.21	*D*_x_ = 1.360 Mg m^−^^3^
Monoclinic, *P*2_1_/*c*	Melting point: 419(1) K
Hall symbol: -P 2ybc	Mo *K*α radiation, λ = 0.71073 Å
*a* = 5.0511 (3) Å	Cell parameters from 8408 reflections
*b* = 10.0219 (7) Å	θ = 1–27.5°
*c* = 20.0840 (12) Å	µ = 0.10 mm^−^^1^
β = 90.107 (6)°	*T* = 150 K
*V* = 1016.67 (11) Å^3^	Block, colorless
*Z* = 4	0.20 × 0.18 × 0.13 mm

### Data collection

**Table:**

Bruker–Nonius KappaCCD area-detector diffractometer	2236 independent reflections
Radiation source: fine-focus sealed tube	1662 reflections with *I* > 2σ(*I*)
graphite	*R*_int_ = 0.048
Detector resolution: 9.091 pixels mm^-1^	θ_max_ = 27.5°, θ_min_ = 2.0°
φ and ω scans	*h* = −6→6
Absorption correction: integration (Coppens, 1970)	*k* = −13→13
*T*_min_ = 0.979, *T*_max_ = 0.987	*l* = −26→22
8320 measured reflections	

### Refinement

**Table:**

Refinement on *F*^2^	Primary atom site location: structure-invariant direct methods
Least-squares matrix: full	Secondary atom site location: difference Fourier map
*R*[*F*^2^ > 2σ(*F*^2^)] = 0.048	Hydrogen site location: inferred from neighbouring sites
*wR*(*F*^2^) = 0.112	H-atom parameters constrained
*S* = 1.13	*w* = 1/[σ^2^(*F*_o_^2^) + (0.0363*P*)^2^ + 0.36*P*] where *P* = (*F*_o_^2^ + 2*F*_c_^2^)/3
2236 reflections	(Δ/σ)_max_ < 0.001
136 parameters	Δρ_max_ = 0.18 e Å^−^^3^
0 restraints	Δρ_min_ = −0.20 e Å^−^^3^

### Special details

**Table:**

Geometry. All e.s.d.'s (except the e.s.d. in the dihedral angle between two l.s. planes) are estimated using the full covariance matrix. The cell e.s.d.'s are taken into account individually in the estimation of e.s.d.'s in distances, angles and torsion angles; correlations between e.s.d.'s in cell parameters are only used when they are defined by crystal symmetry. An approximate (isotropic) treatment of cell e.s.d.'s is used for estimating e.s.d.'s involving l.s. planes.
Refinement. Refinement of *F*^2^ against ALL reflections. The weighted *R*-factor *wR* and goodness of fit *S* are based on *F*^2^, conventional *R*-factors *R* are based on *F*, with *F* set to zero for negative *F*^2^. The threshold expression of *F*^2^ > σ(*F*^2^) is used only for calculating *R*-factors(gt) *etc*. and is not relevant to the choice of reflections for refinement. *R*-factors based on *F*^2^ are statistically about twice as large as those based on *F*, and *R*- factors based on ALL data will be even larger.

### Fractional atomic coordinates and isotropic or equivalent isotropic displacement parameters (Å^2^)

**Table:**

	*x*	*y*	*z*	*U*_iso_*/*U*_eq_	
O1	0.0482 (3)	−0.08173 (13)	0.07156 (6)	0.0440 (4)	
O2	−0.2693 (3)	0.06464 (14)	0.04647 (6)	0.0443 (4)	
H2	−0.1915	0.0654	0.0107	0.053*	
O3	0.1138 (2)	0.13027 (12)	0.19230 (6)	0.0398 (3)	
O4	0.7706 (3)	−0.03563 (14)	0.44558 (7)	0.0477 (4)	
C1	−0.1507 (3)	−0.01793 (17)	0.08665 (8)	0.0323 (4)	
C2	−0.2850 (3)	−0.03301 (19)	0.15227 (8)	0.0355 (4)	
H2A	−0.4267	−0.0977	0.1478	0.043*	
H2B	−0.3645	0.0517	0.1644	0.043*	
C3	−0.1025 (3)	−0.07690 (17)	0.20779 (8)	0.0317 (4)	
H3A	−0.0091	−0.1569	0.1941	0.038*	
H3B	−0.2075	−0.0989	0.2467	0.038*	
C4	0.0958 (3)	0.02952 (16)	0.22605 (8)	0.0297 (4)	
C5	0.2686 (3)	0.00865 (16)	0.28458 (8)	0.0287 (4)	
C6	0.2583 (3)	−0.10711 (17)	0.32209 (8)	0.0333 (4)	
H6	0.1367	−0.1730	0.3108	0.040*	
C7	0.4243 (3)	−0.12673 (18)	0.37606 (9)	0.0363 (4)	
H7	0.4169	−0.2059	0.4002	0.044*	
C8	0.6011 (3)	−0.02784 (18)	0.39356 (8)	0.0343 (4)	
C9	0.6134 (4)	0.09014 (18)	0.35680 (9)	0.0365 (4)	
H9	0.7319	0.1570	0.3688	0.044*	
C10	0.4502 (3)	0.10717 (17)	0.30291 (9)	0.0339 (4)	
H10	0.4605	0.1855	0.2782	0.041*	
C11	0.7813 (5)	−0.1587 (2)	0.48107 (11)	0.0561 (6)	
H11A	0.6127	−0.1758	0.5013	0.067*	
H11B	0.9151	−0.1535	0.5150	0.067*	
H11C	0.8233	−0.2297	0.4508	0.067*	

### Atomic displacement parameters (Å^2^)

**Table:**

	*U*^11^	*U*^22^	*U*^33^	*U*^12^	*U*^13^	*U*^23^
O1	0.0500 (8)	0.0510 (8)	0.0311 (7)	0.0157 (7)	0.0057 (6)	−0.0004 (6)
O2	0.0502 (8)	0.0558 (8)	0.0268 (6)	0.0147 (7)	0.0026 (6)	0.0017 (6)
O3	0.0442 (7)	0.0337 (7)	0.0413 (7)	0.0016 (6)	−0.0026 (6)	0.0066 (6)
O4	0.0528 (8)	0.0466 (8)	0.0436 (8)	−0.0063 (7)	−0.0184 (6)	0.0049 (6)
C1	0.0358 (9)	0.0341 (9)	0.0268 (8)	−0.0008 (8)	−0.0027 (7)	−0.0039 (7)
C2	0.0317 (8)	0.0441 (10)	0.0307 (9)	−0.0015 (8)	0.0018 (7)	−0.0002 (8)
C3	0.0323 (8)	0.0360 (9)	0.0267 (8)	0.0005 (7)	0.0022 (7)	−0.0010 (7)
C4	0.0303 (8)	0.0295 (9)	0.0293 (8)	0.0062 (7)	0.0066 (7)	−0.0007 (7)
C5	0.0285 (8)	0.0288 (8)	0.0288 (8)	0.0017 (7)	0.0039 (6)	−0.0024 (7)
C6	0.0355 (9)	0.0306 (9)	0.0338 (9)	−0.0057 (7)	0.0001 (7)	−0.0005 (7)
C7	0.0408 (9)	0.0334 (9)	0.0348 (9)	−0.0025 (8)	−0.0015 (8)	0.0046 (7)
C8	0.0343 (9)	0.0381 (10)	0.0304 (9)	0.0015 (8)	−0.0025 (7)	−0.0023 (7)
C9	0.0372 (9)	0.0320 (9)	0.0402 (10)	−0.0068 (8)	−0.0040 (8)	−0.0032 (8)
C10	0.0364 (9)	0.0288 (9)	0.0364 (9)	0.0001 (7)	0.0031 (7)	0.0006 (7)
C11	0.0659 (14)	0.0541 (13)	0.0481 (12)	−0.0033 (11)	−0.0225 (10)	0.0106 (10)

### Geometric parameters (Å, °)

**Table:**

O1—C1	1.230 (2)	C5—C6	1.384 (2)
O2—C1	1.301 (2)	C5—C10	1.396 (2)
O2—H2	0.8201	C6—C7	1.383 (2)
O3—C4	1.220 (2)	C6—H6	0.9300
O4—C8	1.352 (2)	C7—H7	0.9301
O4—C11	1.425 (2)	C8—C7	1.379 (2)
C1—C2	1.491 (2)	C8—C9	1.395 (2)
C2—C3	1.511 (2)	C9—H9	0.9300
C2—H2A	0.9700	C10—C9	1.370 (2)
C2—H2B	0.9699	C10—H10	0.9299
C3—H3A	0.9700	C11—H11A	0.9600
C3—H3B	0.9701	C11—H11B	0.9600
C4—C3	1.508 (2)	C11—H11C	0.9600
C5—C4	1.478 (2)		
			
C1—O2—H2	109.2	C10—C5—C4	119.76 (15)
C8—O4—C11	117.38 (15)	C7—C6—C5	121.48 (16)
O1—C1—O2	123.60 (15)	C7—C6—H6	119.3
O1—C1—C2	122.58 (16)	C5—C6—H6	119.2
O2—C1—C2	113.77 (15)	C8—C7—C6	119.26 (16)
C1—C2—C3	113.83 (14)	C8—C7—H7	120.4
C1—C2—H2A	108.8	C6—C7—H7	120.3
C3—C2—H2A	108.8	O4—C8—C7	124.36 (17)
C1—C2—H2B	108.8	O4—C8—C9	115.40 (16)
C3—C2—H2B	108.7	C7—C8—C9	120.24 (16)
H2A—C2—H2B	107.6	C10—C9—C8	119.74 (16)
C4—C3—C2	112.19 (15)	C10—C9—H9	120.1
C4—C3—H3A	109.3	C8—C9—H9	120.2
C2—C3—H3A	109.2	C9—C10—C5	120.94 (16)
C4—C3—H3B	109.2	C9—C10—H10	119.5
C2—C3—H3B	109.1	C5—C10—H10	119.6
H3A—C3—H3B	107.9	O4—C11—H11A	109.5
O3—C4—C5	120.98 (15)	O4—C11—H11B	109.5
O3—C4—C3	120.04 (15)	H11A—C11—H11B	109.5
C5—C4—C3	118.98 (14)	O4—C11—H11C	109.4
C6—C5—C10	118.33 (15)	H11A—C11—H11C	109.5
C6—C5—C4	121.91 (15)	H11B—C11—H11C	109.5

### Hydrogen-bond geometry (Å, °)

**Table:**

*D*—H···*A*	*D*—H	H···*A*	*D*···*A*	*D*—H···*A*
O2—H2···O1^i^	0.82	1.81	2.628 (3)	173
C6—H6···O3^ii^	0.93	2.34	3.247 (3)	164
C11—H11B···O4^iii^	0.96	2.60	3.328 (3)	133
C3—H3B···Cg1^iv^	0.97	2.74	3.591 (3)	146

Symmetry codes: (i) −*x*, −*y*, −*z*; (ii) −*x*, *y*−1/2, −*z*+1/2; (iii) −*x*+2, −*y*, −*z*+1; (iv) *x*+1, *y*, *z*.

## References

[bb1] Allen, F. H., Kennard, O., Watson, D. G., Brammer, L., Orpen, A. G. & Taylor, R. (1987). *J. Chem. Soc. Perkin Trans. 2*, pp. S1–19.

[bb2] Altomare, A., Cascarano, G., Giacovazzo, C., Guagliardi, A., Burla, M. C., Polidori, G. & Camalli, M. (1994). *J. Appl. Cryst.* **27**, 435.

[bb3] Coppens, P. (1970). *Crystallographic Computing*, edited by F. R. Ahmed, S. R. Hall & C. P. Huber, pp. 255–270. Copenhagen: Munksgaard.

[bb4] Hashem, A. I., Youssef, A. S. A., Kandeel, K. A. & Abou-Elmangd, W. S. I. (2007). *Eur. J. Med. Chem.* **42**, 934–939. 10.1016/j.ejmech.2006.12.032 17321008

[bb5] Hooft, R. W. W. (1998). *COLLECT* Nonius BV, Delft, The Netherlands.

[bb6] Husain, A., Khan, M. S. Y., Hasan, S. M. & Alam, M. M. (2005). *Eur. J. Med. Chem.* **40**, 1394–1404. 10.1016/j.ejmech.2005.03.012 15878219

[bb7] Otwinowski, Z. & Minor, W. (1997). *Methods in Enzimology*, Vol. 276, *Macromolecular Crystallography*, Part A, edited by C. W. Carter Jr & R. M. Sweet, pp. 307–326. New York: Academic Press.

[bb8] Sheldrick, G. M. (2008). *Acta Cryst.* A**64**, 112–122. 10.1107/S0108767307043930 18156677

[bb9] Spek, A. L. (2003). *J. Appl. Cryst.* **36**, 7–13.

